# Losing the Big Picture: How Religion May Control Visual Attention

**DOI:** 10.1371/journal.pone.0003679

**Published:** 2008-11-12

**Authors:** Lorenza S. Colzato, Wery P. M. van den Wildenberg, Bernhard Hommel

**Affiliations:** 1 Leiden University, Cognitive Psychology Unit & Leiden Institute for Brain and Cognition, Leiden, The Netherlands; 2 Psychology Department, Universiteit van Amsterdam, Amsterdam Center for the Study of Adaptive Control in Brain and Behaviour (ACACia), Amsterdam, The Netherlands; University of Granada, Spain

## Abstract

Despite the abundance of evidence that human perception is penetrated by beliefs and expectations, scientific research so far has entirely neglected the possible impact of religious background on attention. Here we show that Dutch Calvinists and atheists, brought up in the same country and culture and controlled for race, intelligence, sex, and age, differ with respect to the way they attend to and process the global and local features of complex visual stimuli: Calvinists attend less to global aspects of perceived events, which fits with the idea that people's attentional processing style reflects possible biases rewarded by their religious belief system.

## Introduction

Perceiving our world is an active process. We do not passively register the objects and events we encounter but explore and investigate them, attend to features and characteristics we find interesting and ignore those that we do not. As emphasized by the New Look perspective to human perception [1], this suggests that our perception and attention reflect our moods, needs, expectations, and beliefs. Recent research has extended the list of possible factors to culture. Increasing evidence suggests, for instance, that people growing up in the North American culture are less sensitive to contextual cues and show a more analytic cognitive style (i.e., they pay more attention to local features of objects and events) than people growing up in an Asian culture, who exhibit a more holistic style [2], [3]. Holistic and analytic processing styles can be induced even within the same population by having people work through tasks that draw attention to either personal interdependence (e.g., by instructing participants to circle all relational pronouns, such as “we”, “our”, or “us”, in a text) or independence (by having them to circle pronouns referring to the self independent of others, such as “I”, “my”, or “me”) [4]. Recent electrophysiological evidence suggests that culturally or experimentally induced attention to the global context versus local detail affects the processing of visual features rather early in the processing stream. In particular, marking independent pronouns yields an enlarged P1 amplitude to local than global targets in a global-local task ([5]; see below) at lateral occipital electrodes (i.e., in the visual cortex), while marking interdependent pronouns has the opposite effect [6].

However, research so far has completely ignored the influence of religion on attentional processing. Given that culture is commonly defined as a system of shared beliefs, values, customs, behaviors, and artifacts, this blind spot is surprising. It is even more surprising if one considers the recent history of increasingly dramatic international and societal conflicts based on apparent incompatibilities between the religious beliefs of social groups and nations and the behavioral implications thereof. If the perception of events would be modulated not only by upbringing and culture but also, or perhaps even mainly, by religious factors, it would seem particularly important to study how they affect perception. Here, we provide evidence that religious belief may systematically bias visual attention.

We investigated whether Calvinists and atheists, brought up in the same country and culture (the Netherlands), differ with respect to the way they attend to and process global and local features of visual stimuli. Cultural (and, possibly, other) differences in perceptual processing and attentional emphasis are assumed to be produced by social practice [7], [8] that provides selective reward for attending to particular stimulus features and adopting particular attentional control settings [5], [6]. We speculate that practicing a religion and being exposed to particular religious practices may lead, among other things, to a chronic bias towards particular attentional control parameters. In particular, our study was inspired by the Dutch neo-Calvinism concept of *sphere sovereignty*, which emphasizes that each sphere or sector of society has its own responsibilities and authorities, and stands equal to other spheres [9], [10]. If Dutch Calvinists, as compared to Dutch atheists, have been rewarded more for adopting a processing style that emphasizes a rather independent view of the self, this would be likely to induce an attentional set that facilitates the processing of the local details [6]. If so, this should affect performance on the global-local task developed by Navon [5], which indexes how fast people can process global and local characteristics of hierarchically constructed visual stimuli (e.g., larger letters made of smaller letters). Typically, this task gives rise to the “global precedence” effect, which means that global features can be processed faster than local features. According to our reasoning, Calvinists as compared to atheists might show a less pronounced, if any, global precedence effect [6], [7].

## Results

The square root of error percentages and median reaction times were analyzed by means of analysis of variance (ANOVA) using Target Level (global vs. local) as within- and Group (Calvinists vs. Atheists) as between-participants factor. The reaction time analysis showed a main effect of Target Level, *F*(1,38) = 215.32, *p* = .0003, *MSE* = 544.632, *η^2^p* = 0.85, which was modified by Group, *F*(1,38) = 8.34, *p* = .006, *MSE* = 544.632, *η^2^p* = 0.18. The main effect indicated global precedence [5]: Global targets were responded to faster than local targets. However, as expected, the size of this effect varied with Group: Calvinists showed a smaller, but still significant, *F*(1,19) = 147.69, *p* = .0002, *MSE* = 256.092, *η^2^p* = 0.88, global precedence effect than Atheists (see Table 1). Error percentages did not reveal any reliable effect, *F's*(1, 30)<1.

**Table 1:** Demographic characteristics, religious behaviour, and performance on globally and locally defined targets.

Variables (SD)	Calvinists	Atheists
*Sample N (M∶F)*	20 (4∶16)	20 (5∶15)
*Age (years)*	21.3 (2.8)	21.7 (2.9)
*Raven IQ*	112.8 (3.5)	115.6 (4.8)
Baptized (or similar)**	20 (0)	0 (0)
Daily prays**	5.6 (1.5)	0 (0)
Weekly church visit**	2.0 (0)	0 (0)
*Global Targets*		
Reaction Times (ms)	361 (11.5)	359 (11.5)
Error Rates (%)	8.8 (2.0)	7.3 (2.0)
*Local Targets*		
Reaction Times (ms)	423 (14.5)	450 (14.5)
Error Rates (%)	6.7 (1.6)	9.6 (1.6)
*Global Precedence*		
Reaction Times (ms)**	62	91
Error Rates (%)	−2.1	2.3

Standard errors are presented within parentheses.

Significant group difference; * *p*<0.05, ** *p*<0.01.

We further tested whether Age and IQ contributed to the effect on the global precedence. An ANOVA with group as independent variable and age and IQ as covariates indicated no such contribution: the effects of the covariates were far form significant, for both *F*<1, and the Group effect remained clearly reliable, *F*(1,36) = 7.73, *p* = .009, *MSE* = 541.697, *η^2^p* = 0.18.

## Discussion

This outcome suggests that religious belief biases the way people attend to and process visual events: Calvinists showed a less pronounced global precedence effect than atheists, indicating that practicing this religion might lead one to attend to more local aspects. True, given the correlational nature of our observation we cannot exclude the possibility that Calvinism is more attractive for people with a more local attentional bias. However, people commonly join religious groups before such biases become obvious (often by birth, following family traditions), which seems to undermine this possibility. As our groups were matched for sex, IQ, age, educational style and socio-economic situation we can rule out an account of our results in these terms. Particularly important was the matching of the age range and educational style. Developmental studies indicated that the global precedence effect is unrelated to general intelligence but changes with age [11].

According to our approach, social experience and procedures (in our case religion), and the selective reward they provide, can induce the emphasis on and higher weighting [12] of socially relevant perceptual features and characteristics of processed events. We speculate that exercising a religion and being exposed to particular religious practices may lead, among other things, to a chronic bias towards particular attentional control parameters. The sphere sovereignty principle underlying Dutch neo-Calvinism has led to a rigorous “pillarization” (segregation) of Dutch society and established the idea that, in a nutshell, everyone should “mind his or her own business”—which among other things inspired a rather liberal policy regarding drug use, abortion, or euthanasia. Calvinists may have learned since early age to focus on local rather than global dimensions, at least as compared to people not sharing their religious practices. In general, we suggest that peoples' attentional processing style reflects possible biases rewarded by their religious belief.

Another possibility is that our results reflect a more general difference between believers and non-believers. Being a believer (totally and/or strongly focusing on one religion), as in the case of our Calvinists, might as such lead to a less pronounced global precedence effect. Even though this possibility would still be consistent with our approach, further research is necessary to get a deeper insight into the responsible processing mechanisms. In the Netherlands it is very difficult to find other comparable religious group to match without losing purity of culture. Most Dutch Catholics (the only other religious community with a sizeable membership) live in Limburg (at the border to Germany) and Brabant (at the border to Belgium). Given the proximity to these other countries and the resulting mix of inhabitants (many Dutch actually live in Germany and Belgium, and many Germans and Belgians live in the Netherlands) it is hard to find a sizeable sample of Catholics not being exposed to another culture on an everyday basis. We therefore plan to investigate religious belief systems outside the Netherlands, including Orthodox Judaism, which emphasizes social solidarity—a condition that might lead to an increase of the global precedence effect.

In sum, given that real-life objects and events are commonly complex and hierarchically structured, so that their perceptual organization and semantic interpretation often hinges on the aspect or level an observer attends to, it seems possible that religious beliefs may indeed lead to different and sometimes discrepant and incompatible interpretations of the same incident. That this can happen is a well-known empirical fact but that it can originate in basic automatic visual operations that precede conscious representation is surprising and in some sense worrying—as it seems to work against the scientific ideal that careful observation is sufficient to reach agreements about basic facts and what we consider reality. Our findings are consistent with the New Look on perception [1] in confirming that perceptual processes can be affected by the cognitive states of the observer. To some degree they challenge, however, previous claims that culture has an important impact on perception. Even though our findings do not rule out this possibility, they show that religion makes a difference even if culture is controlled for. Given that previous reports on culture-related differences did not control for religion [2], [3], it is possible that religious differences are sufficient to account for the available evidence.

## Materials and Methods

### Participants

We tested 40 young healthy adults (all students from Leiden University, Institute for Psychological Research), who participated for partial fulfillment of course credit or a financial reward. They constituted two experimental groups: Calvinists (all members of the Calvinistic corps of Leiden University) and Atheists. All participants were matched for race (100% Caucasian), Culture (100% Dutch), age, sex, and IQ (measured by Raven's Standard Progressive Matrices)—see Table 1 for demographic data and religious behavior. All Calvinists and Atheists were educated in The Netherlands following the same educational style and institution type (VWO), and reported similar social-economical background. Written informed consent was obtained from all participants after the nature of the study was explained to them; the protocol was approved by the institutional review board (Leiden University, Institute for Psychological Research), which approved the remuneration arrangements of 10 Euro.

### Apparatus and stimuli

Responses were made by pressing the “Z” or “?” of the QWERTY computer keyboard with the left and right index finger, respectively. The target stimuli were adopted from Huizinga, Dolan and van der Molen [11], and consisted of geometric figures. Larger (global) rectangles/squares consisted of smaller (local) rectangles or squares. Global stimuli (i.e., squares or rectangles; 93×93 pixels or 93×189 pixels respectively) were composed of many smaller “local” stimuli (i.e., squares or rectangles; 21×21 pixels or 8×46 pixels respectively). The space between the local elements of a stimulus was 3 pixels. A global square consisted of 16 small squares or 8 small rectangles; a global rectangle consisted of 32 small squares or 16 small rectangles.

### Procedure and design

All participants were tested individually and completed the intelligence test and the Local-Global Task.

Individual IQ was determined by means of a 30-min reasoning-based intelligence test (Raven's Standard Progressive Matrices: SPM [13]). Each item of this test consists of a pattern or sequence of a diagrammatic puzzle with one piece missing. The task is to complete the pattern or sequence by choosing the correct missing piece from a list of options. The items are getting more difficult as the test taker proceeds through the test. The SPM assesses the individual's ability to create perceptual relations and to reason by analogy independent of language and formal schooling; it is a standard, widely-used test to measure Spearman's g factor and fluid intelligence in particular.

In the Local-Global Task (cf., [11]), participants responded to randomly presented rectangles or squares by pressing a left or right response button, respectively. Larger (global) rectangles/squares consisted of smaller (local) rectangles or squares. Participants responded to the local figure in one block and to the global figure in another (blocks 1 and 2, in randomized order; 30 practice trials and 100 experimental trials per block). A cue indicated to which dimension (global or local) the participants should respond. Cues that signalled the global (local) dimension consisted of a big (small) square, presented at one side of the target stimulus, and a big (small) rectangle, presented at the other side of the target stimulus. The color of cues and target was red. They remained on the screen until a response was given. Participants had 3500 ms to respond. The time interval between presentation of the cue and of the target stimulus was 500 ms. The interval between the response and the presentation of the cue was fixed at 1000 ms. The main dependent variable was the median response latency to local and global targets.
